# X-ray crystal structures *Corynebacterium diphtheriae* sortases

**DOI:** 10.1063/4.0001117

**Published:** 2025-10-27

**Authors:** Jerzy Osipiuk, Poonam Kamari, HyLam Ton-That, Hung Ton-That, Andrzej Joachimiak

**Affiliations:** 1 Argonne National Laboratory, Lemont, IL, USA; 2 University of CHicago, Chicago, IL, USA; 3 University of California Los Angeles, Los Angeles, CA, USA; 4 University of California, Irvine, CA, USA; 5 University of Chicago, Chicago, IL, USA

## Abstract

Pathogenic members of the genus Corynebacterium cause a wide range of serious infections in humans including diphtheria. Adhesion to host cells is a crucial step during infection. In Corynebacterium diphtheriae, adhesion is mediated by filamentous structures called pili or fimbriae that are covalently attached to the bacterial cell wall. Pilus assembly proceeds by transpeptidation reactions catalyzed by sortases, followed by covalent anchoring of the filament in the peptidoglycan layer.

There are 6 sortases in C. diphtheriae, SrtA, SrtB, SrtC, SrtD, SrtE and SrtF. Five of these proteins are devoted to the assembly of three distinct types of pilus fibres: SrtA for the SpaA-type pilus, SrtB/SrtC for the SpaD-type pilus, and SrtD/SrtE for the SpaH-type pilus. SrtF, the so-called housekeeping sortase, catalyses the cell wall anchoring of pilin monomers as well as pili, but it does not polymerize pilins.

We have previously determined structures of the SrtA and SrtF sortases and deposited to Protein Data Bank (PDB). There are no structures of remaining four C. diphtheriae sortases available. Also, biological data for these proteins are sparse. The presence of two sortase pairs involved in the SpaD-and SpaH-type pilus assembly is intriguing. The SrtB/SrtC sortases are able to polymerize SpaDF pili consisting of SpaD and SpaF proteins. However, only SrtB is capable of incorporation of SpaE accessory protein into the SpaD-type pili. There is even less data for SrtD/SrtE pair (40% amino acid identity between these proteins). It was shown that both proteins participate in formation of SpaHIG pili (SpaH-type pili including two accessory proteins SpaI and SpaG). Deletion of either one of sortases results in significantly slower polymerization of SpaH-type pili. No clear difference between SrtD and SrtE was reported.

We crystallized and solved the high-resolution structures of four remaining C. diphtheriae sortases: SrtB, SrtC, SrtD and SrtE. The structures, with exception of SrtD, closely resemble Alphafold prediction models. We did not find significant differences between SrtB and SrtC sortase main bodies and active centers. The major difference between SrtB and SrtC is the presence SrtB extended proline-rich 18-residue loop replacing short 3-residue fragment in the SrtC. We think this loop is indirectly involved in the SrtB activity. Also, for SrtD and SrtE sortase pair, we did not find significant differences in core structures and active centers. Nevertheless, we found a unique conformation of the N-terminal helix not predicted by Alphafold models. The helix of the experimental structure is rotated by 180 degrees relatively to the protein main body. We do not exclude possibility that it is a result of crystal packing. Nevertheless, it shows mobility /flexibility of this SrtD region. We think that our structural data and mutagenesis of sortases will help to elucidate differences in sortase pairs and will help understand their biological purpose.

